# Supplementary material for: Dissipative phases across the superconductor-to-insulator transition
Source: Sci Rep. 2016 Oct 27;6:35834. doi: 10.1038/srep35834 (PMC5081520; doi:10.1038/srep35834)
Supplement: Supplementary Information [file srep35834-s1.pdf]

Supplementary materials for

Dissipative phases across the superconductor-to-insulator transition

F. Couëdo, O. Crauste, A.A. Drillien, V. Humbert, L. Bergé, C.A. Marrache-Kikuchi* and L. Dumoulin

CSNSM, Univ. Paris-Sud, CNRS/IN2P3, Université Paris-Saclay, 91405 Orsay, France

*Corresponding author (email: claire.marrache@csnsm.in2p3.fr)

This supplementary material includes:

- Supplementary Text
- Figure S1

Following the method described in [S1], we analyzed the resistance close to the superconducting transition using the formula:

$$R(T) = \frac{1}{1/R_{\mathrm{ref}} + \Delta \mathrm{G(T)}}$$

R_{ref} is chosen at high temperature, typically around 10K. $\Delta \mathrm{G}(T)$ is the quantum correction to the conductance and includes:

- the weak localization and the Coulomb interaction term $\Delta G^{\mathrm{WL}} + \Delta G^{\mathrm{IEE}} = G_{00}\,\mathrm{A}\ln(\frac{T\tau k_{\mathrm{B}}}{\hbar})$, where $G_{00} = \frac{e^2}{2\pi^2\hbar}$, the coefficient A reflects the weight of the localization and τ is the elastic mean free path

- the 2D Aslamazov-Larkin term $\Delta G^{\mathrm{AL}} = \frac{e^2}{16\hbar}\varepsilon^{-1}$ describing the formation of fluctuating Cooper pairs above T_{c0}, where $\varepsilon = \frac{T - T_{\mathrm{c0}}}{T_{\mathrm{c0}}}$

- the correction $\Delta G^{\mathrm{DOS}} = G_{00}\ln[\frac{\ln(T_{\mathrm{c0}}/T)}{\ln(\tau k_{\mathrm{B}} T_{\mathrm{c0}}/\hbar)}]$ due to the reduction of single electron density of states by the formation of the fluctuating Cooper pairs

- the Maki-Thompson correction $\Delta G^{MT} = \frac{e^2}{8\hbar}\frac{1}{\varepsilon - \delta}\ln(\varepsilon/\delta)$ arising from the coherent scattering of the electrons forming a Cooper pair on impurities, δ is the pair-breaking parameter

The fitting parameters here are the coefficient A, T_{c0} and δ.

Figure S1 shows the result of this analysis for the 23-nm-thick a-$Nb_{13.5}Si_{86.5}$ film, annealed at θ_{ht} = 110°C. We obtained A = 2.1, consistent with previous results on disordered thin films [S1]. δ= 0.03 matches the expected value (δ= 0.03), taking into account the normal state resistance R_{N} = 646 Ω [S2]. Finally, the value of $T_{\mathrm{c0}} = 0.058\ \mathrm{K}$ agrees well with the one defined by the maximum of the derivative at low temperature ($T_{\mathrm{c0}} = 0.06\ \mathrm{K}$).

[S1] Sacepe, B. and Chapelier, C. and Baturina, T. I. and Vinokur, V. M. and Baklanov, M. R. and Sanquer, M. Pseudogap in a thin film of a conventional superconductor. Nature Comm. **1,** 140 (2010).

[S2] Baturina, T. I., Postolova, S. V., Mironov, A. Y., Glatz, A., Baklanov, M. R., and Vinokur, V. M. Superconducting phase transitions in ultrathin TiN films. *EPL (Europhysics Letters)* ***97*,** 17012 (2012).

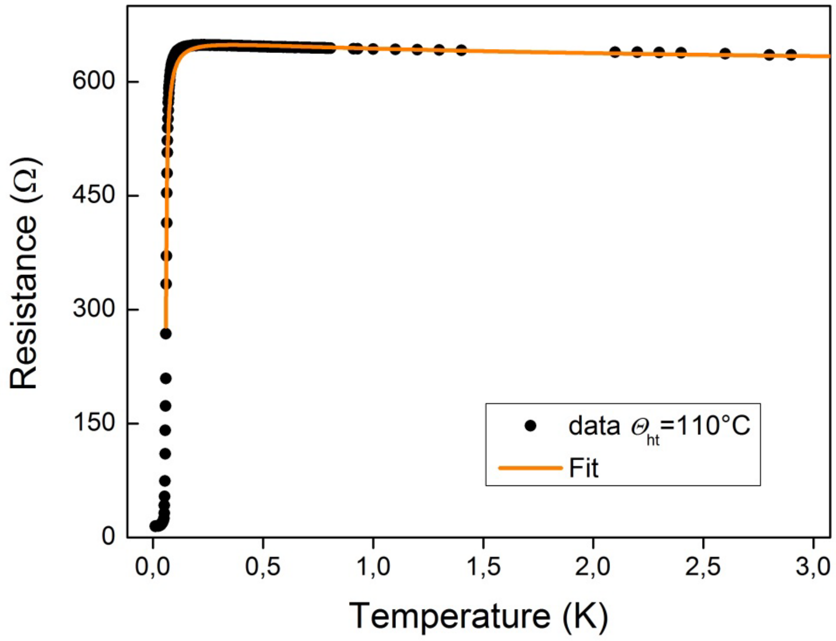

Supplementary Figure S1. Sheet resistance as a function of temperature for the 23-nm-thick a-$Nb_{13.5}Si_{86.5}$ film at the annealing temperature θ_{ht} = 110° C (Black). The orange curve shows the fitting of the quantum corrections with $A = 2.1$, $T_{\mathrm{c0}} = 0.058\ \mathrm{K}$ and $\delta =\ 0.03$.
